# Recombinant Factor VIIa Reduces Bleeding after Blunt Liver Injury in a Pig Model of Dilutional Coagulopathy under Severe Hypothermia

**DOI:** 10.1371/journal.pone.0113979

**Published:** 2015-06-22

**Authors:** Henri M. H. Spronk, Till Braunschweig, Rolf Rossaint, Dirk C. Wüst, Rene van Oerle, Brian Lauritzen, Rene Tolba, Oliver Grottke

**Affiliations:** 1 Laboratory for Clinical Thrombosis and Haemostasis, Department of Internal Medicine, Cardiovascular Research Institute Maastricht, Maastricht University, Maastricht, The Netherlands; 2 Department of Pathology, RWTH Aachen University Hospital, Aachen, Germany; 3 Department of Anaesthesiology, RWTH Aachen University Hospital, Aachen, Germany; 4 Novo Nordisk A/S, Novo Nordisk Park G2P.S, Måløv, Denmark; 5 Institute for Laboratory Animal Science, RWTH Aachen University Hospital, Aachen, Germany; Emory University School of Medicine, UNITED STATES

## Abstract

**Background:**

Recombinant factor VIIa (rFVIIa) is registered for use in haemophilia with inhibitors and other rare bleeding disorders, but has also been used in various other clinical conditions to terminate life-threatening bleeding. Underlying conditions (e.g. coagulopathy) and dosing may affect treatment efficacy. The objective of the present study was to evaluate the impact of increasing doses of rFVIIa on blood loss and coagulation assays in haemodiluted and hypothermic pigs undergoing blunt liver injury.

**Methods:**

A grade III blunt liver injury was induced in 28 pigs after 70% haemodilution and cooling to 32.6–33.4°C. Ten minutes after trauma, animals randomly received placebo or 90, 180 or 360 μg/kg rFVIIa. Global coagulation parameters, thromboelastometry (TEM) and plasma thrombin generation (TG) were determined at different time points during the observation period of 120 minutes.

**Results:**

Total blood loss was significantly lower following 90 μg/kg rFVIIa (1206 [1138–1470] mL) relative to placebo (2677 [2337–3068] mL; *p*<0.05), with no increased effect with higher dose levels of rFVIIa. Following trauma and haemodilution, coagulation was impaired relative to baseline in both TEM and TG analysis. At 60 and 120 minutes after trauma, TEM variables improved in the rFVIIa-treated animals compared with the placebo group. Similarly, rFVIIa improved coagulation kinetics in TG. As was observed with blood loss, no significant effect between different rFVIIa dose levels was found in TEM or TG. Macro- and microscopic post-mortem examination did not reveal any signs of thromboembolic events.

**Conclusion:**

Early administration of 90 μg/kg rFVIIa reduced blood loss in pigs undergoing blunt liver injury even after severe haemodilution and hypothermia, with no further effect of higher dose levels. Coagulation assays showed impaired coagulation in coagulopathic animals, with a dose-independent improvement in animals treated with rFVIIa.

## Introduction

Trauma is often associated with coagulopathy, which complicates the control of bleeding and leads to a four-fold increase in the risk of mortality [1,2]. Coagulopathy of trauma has multiple causes, including tissue injury, shock, hypothermia, haemodilution, inflammation and acidosis [1,2]. The exact mechanisms by which these processes contribute to coagulopathy remain unclear, but growing evidence suggests that coagulation proteases are involved, along with anticoagulant and fibrinolytic processes [1,3].

Therapy with purified or recombinant coagulation factors, such as fibrinogen, prothrombin complex concentrates (PCCs) and recombinant activated coagulation factor VII (rFVIIa, NovoSeven, Novo Nordisk, Denmark), has been shown to reduce severe bleeding and may thus be used as an approach to restore coagulation in traumatic injury complicated by coagulopathy [4–6]. rFVIIa was initially developed to treat haemophilia patients with inhibitory antibodies [7] and approved indications for its use have now been expanded to include other rare bleeding disorders. However, rFVIIa has also been used outside of licensed indications in the treatment of various other clinical conditions; while a large randomised trial failed to demonstrate a clinical effect of rFVIIa on trauma outcome [8], rFVIIa has been used to stop life-threatening bleeding that is refractory to surgical and haemostatic approaches [9].

The potential effect of rFVIIa may be affected by dosing regimen and the presence of severe haemodilution, hypothermia and acidosis [10–12]. Furthermore, the use of pro-coagulants such as rFVIIa and PCCs in trauma may potentially induce thrombosis or disseminated intravascular coagulation (DIC). A recent study with PCCs using the same porcine liver-bleeding model as that to be reported in the current paper indicated that, while PCCs are effective in attenuating bleeding, there may be a relatively narrow therapeutic window in this setting. Increasing the PCC dose from 35 to 50 IU/kg resulted in thrombosis in all animals, and 44% of animals developed a DIC-like syndrome [13].

In the present study, we investigated the efficacy and safety of increasing doses of rFVIIa in haemodiluted and hypothermic pigs by measuring blood loss after blunt liver injury. Furthermore, the effect of rFVIIa was monitored using a panel of coagulation assays in order to establish a correlation between in vivo and ex vivo effects.

## Methods

### Animals and Anaesthesia

Experiments were performed in 28 male German land-race pigs (body weight 30–36 kg) in accordance with German legislation governing animal studies and following the Principles of Laboratory Animal Care. Official permission (No 8.87–51.04.20.09.346) was granted from the appropriate government office (Landesamt für Natur, Umwelt und Verbraucherschutz Nordrhein-Westfalen, Recklinghausen, Germany) for animal care and use. All animals were housed in ventilated rooms and allowed to acclimatise to their surroundings for a minimum of 5 days before surgery. Prior to the surgical procedure, the pigs fasted overnight and water was provided.

As a pre-medication, all pigs received an intramuscular (i.m.) injection of 4 mg/kg azaperone (Stresnil, Janssen, Neuss, Germany) and 0.1 mg/kg i.m. atropine (Atropinsulfate, B. Braun, Melsungen, Germany). Anaesthesia was induced by intravenous injection of 3 mg/kg propofol (Disoprivan, AstraZeneca, Wedel, Germany) followed by orotracheal intubation. The animals were ventilated at 16–22 breaths/minute and a tidal volume of 8 mL/kg to keep the end-tidal partial pressure of carbon dioxide (pCO2) between 36 and 42 mmHg. Anaesthesia was maintained using isoflurane (1%) and a continuous infusion of fentanyl at 3–4 μg/kg/hour at an inspiratory oxygen fraction of 1.0. Initially, Ringer’s lactate solution (RL, Fresenius, Bad Homburg, Germany) was infused at 4 mL/kg/hour, and the infusion rate was increased to 10 mL/kg/hour after laparotomy until infliction of trauma. To achieve hypothermia of 32.6–33.4°C, room-temperature solutions were used for infusion. The use of a warming blanket prevented further temperature decreases.

Temperature, tail pulse oximetry, electrocardiogram and arterial and central venous pressure were monitored using femorally introduced catheters connected to a standard anaesthesia monitor (AS/3, Datex Ohmeda, Helsinki, Finland).

### Surgical procedures

Two 8.5 Fr catheters were implanted in the left and right jugular veins for insertion of a pulmonary artery catheter and for volume replacement. Subsequently, a midline laparotomy with splenectomy and cystostomy was performed. The intravascular volume was diluted by substituting approximately 70% of the estimated blood volume with hydroxyethylstarch HES 130/0.4 (Voluven, Fresenius, Bad Homburg, Germany) and RL at a ratio of 1:1. Blood was withdrawn at a rate of 100 mL/minute. Blood volume was replaced as soon as the blood pressure dropped to 40–50% of the baseline value and supplementation was continued until the blood pressure had returned to 80% of the baseline value. To prevent early death from anaemia, the collected blood was processed (Cell Saver 5, Haemonetics, Braintree, MA, USA) and the red blood cells were re-transfused (20 mL/kg) over 10 minutes. A reproducible grade III blunt liver injury was induced using a custom-made instrument with a force of 229–303 N [14]. The investigator who performed the liver injury procedure was blinded to the rFVIIa infusion condition. Five minutes after the induction of injury, all animals received RL as a fluid bolus of 35 mL/kg, followed by continuous infusion at 40 mL/kg/hour until the end of the experiment. The 28 animals were randomised 1:1:1:1 using sealed envelopes to receive normal saline solution (controls, n = 7), 90 μg/kg rFVIIa (rFVIIa 90, n = 7), 180 μg/kg rFVIIa (rFVIIa 180, n = 7) or 360 μg/kg rFVIIa (rFVIIa 360, n = 7) 10 minutes after injury. rFVIIa was given in a small volume at a concentration of 600 μg/mL.

The animals were observed for a total period of 120 minutes after injury. Animals surviving for the entire observation period were euthanised using an overdose of pentobarbital. Immediately after euthanasia, the abdomen was reopened, the vena cava was clamped cranially to the liver, and the intra-peritoneal blood was collected to determine the total blood loss after injury.

### Blood sampling and analytical methods

Blood samples for arterial blood gas analysis, whole blood cell count, thromboelastometry and plasma preparation were collected at the following time points: after splenectomy (baseline), at the end of haemodilution, and at 10, 60 and 120 minutes after liver injury. Haemoglobin concentration, pH, partial pressure of oxygen and pCO2 were measured using a blood gas analyser (ABL500, Radiometer, Brønshøj, Denmark). Prothrombin time (PT), activated partial thromboplastin time (aPTT) and fibrinogen concentration were determined by standard laboratory methods using the appropriate kits from Dade Behring (Marburg, Germany) on an MC 4 plus steel-ball coagulometer (Merlin Medical, Lemgo, Germany); the fibrinogen concentration was measured using the Clauss assay. Thrombin–antithrombin complexes (TAT) were quantified by ELISA (Enzygnost, Dade Behring, Marburg, Germany).

### Thromboelastometry and thrombin generation

A rotational thromboelastometry analyser (ROTEM, TEM International GmbH, Munich, Germany) was used to perform the ExTem assay on whole blood according to the manufacturer’s recommendations. The coagulation process was activated with tissue factor (Innovin; Dade Behring, Marburg, Germany) at a final dilution of 1: 34 000 and recalcified with 0.2 M CaCl_2_. The following parameters were derived from the measurement: clotting time (CT, seconds), clot formation time (CFT, seconds) and maximum clot firmness (MCF, mm). To prevent artificial contact activation, blood sampling tubes contained corn trypsin inhibitor (Haemotologic Technologies Inc., Essex Junction, VT, USA) at a final concentration of 100 μg/mL.

Thrombin generation (TG) was measured using the Calibrated Automated Thrombogram (CAT; Thrombinoscope B.V., Maastricht, The Netherlands) using platelet-poor plasma as described previously, using final concentrations of 0.25 or 1 pM tissue factor (TF) and 4 μM phospholipids [15]. To correct for inner-filter effects and substrate consumption, the results from each thrombin generation analysis were calibrated against the fluorescence curve obtained from the same plasma with a fixed amount of calibrator (Thrombin Calibrator, Thrombinoscope B.V., Maastricht, The Netherlands). Fluorescence was measured in an Ascent Reader (Thermolabsystems OY, Helsinki, Finland) equipped with a 390/460 nm filter set, and thrombin generation curves were calculated using Thrombinoscope software, Version 4 (Thrombinoscope B.V., Maastricht, The Netherlands). All coagulation measurements were performed in duplicate.

### Pathological examination

Immediately after post-mortem removal, four internal organs (lungs, heart, liver, kidneys) were fixed in 10% buffered formalin. Injured parts of the liver were cut into slices (3 mm thick) and examined macroscopically and microscopically by a pathologist blinded to the therapy conditions to assess the degree of injury. The pathologist also determined macroscopically and by histology whether (micro-) thrombi had formed in representative tissue sections of all four organs as a means of assessing thromboembolic events. All samples were cross sectioned, representative parts were embedded in paraffin and sections stained, either by haematoxylin and eosin or by a standard Elastica-van Gieson protocol, for histological examination under light microscopy (Eclipse 50i, Nikon, Duesseldorf, Germany). Both staining methods were applied to sections from all of the tissues. Selected sections of lung and liver tissue (regions with a high likelihood of thrombus formation) were immunostained to test for fibrinogen-positive thromboemboli, as described elsewhere (antibodies and detection kits were obtained from DAKO, Glostrup, Denmark) [13].

### Statistics

All statistical analyses were conducted using SAS Version 9.1.3 (SAS Institute Inc., Cary, NC, USA). For graphing, GraphPad Prism (Version 6, GraphPad Software, Inc., CA, USA) was used. Baseline versus haemodilution values were compared using the Wilcoxon test. Multiple t-tests with the Holm-Sidak correction for multiple comparisons were applied for rFVIIa treatment versus non-treatment at 10, 60 and 120 minutes. Data on survival were analysed using the log-rank test. Parameters are presented as the median and inter-quartile ranges (IQR). Statistical tests were two-tailed and *P*<0.05 was defined as significant.

## Results

### Baseline and haemodilution

**Fig 1:**
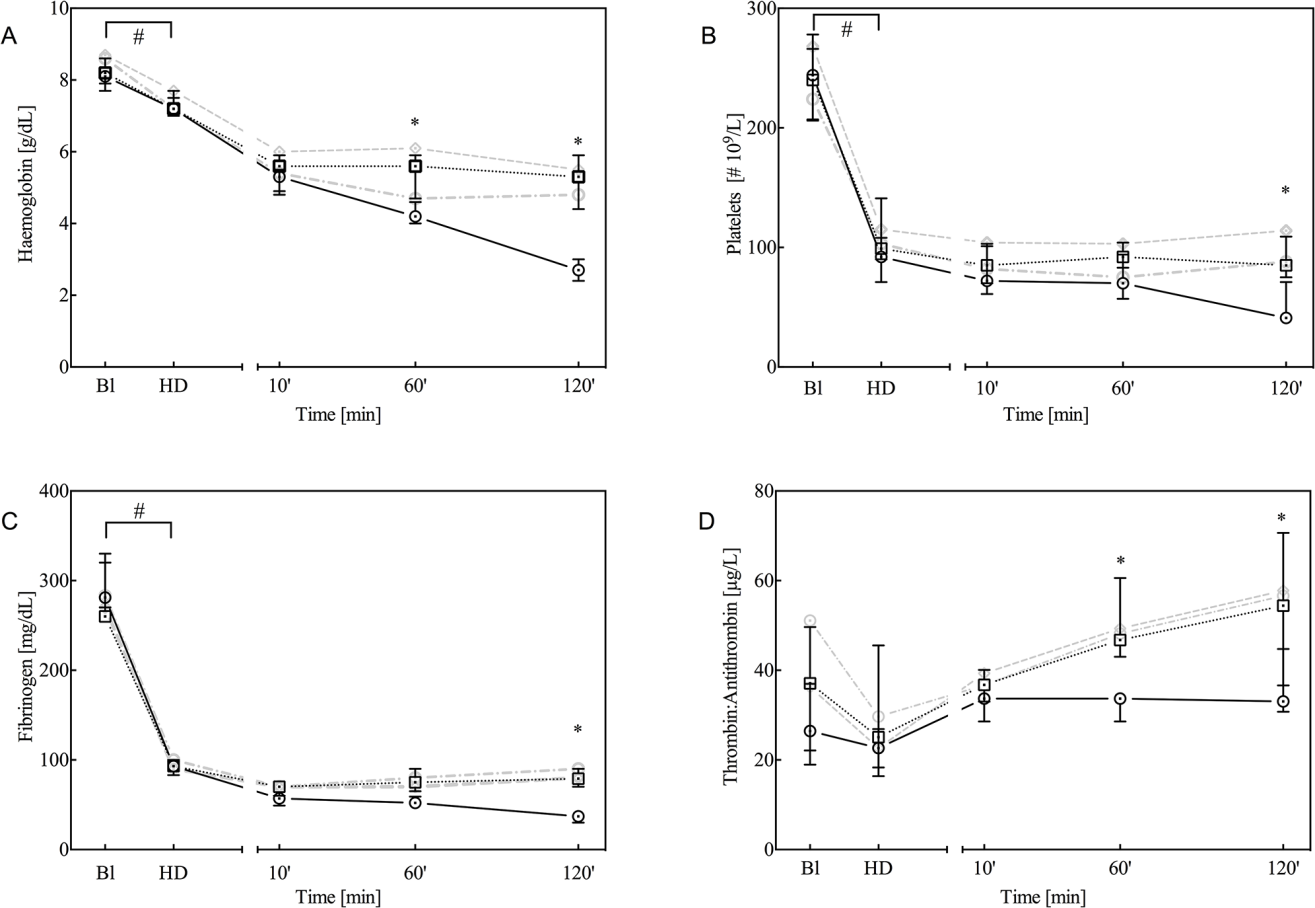
Haemoglobin (A), platelet count (B), fibrinogen (C) and thrombin-antithrombin (TAT; D) levels at baseline (Bl), after haemodilution (HD) and 10, 60 and 120 minutes after trauma. Data are presented as the median with inter-quartile ranges (IQR) for controls (solid black line, n = 7) and animals treated with 90 μg/kg rFVIIa (dotted black line, n = 7). For comparison, data from animals treated with either 180 or 360 μg/kg rFVIIa are presented as grey dotted lines. *P<0.05 control versus rFVIIa; #*P*<0.05 over time between baseline and haemodilution.

**Fig 2:**
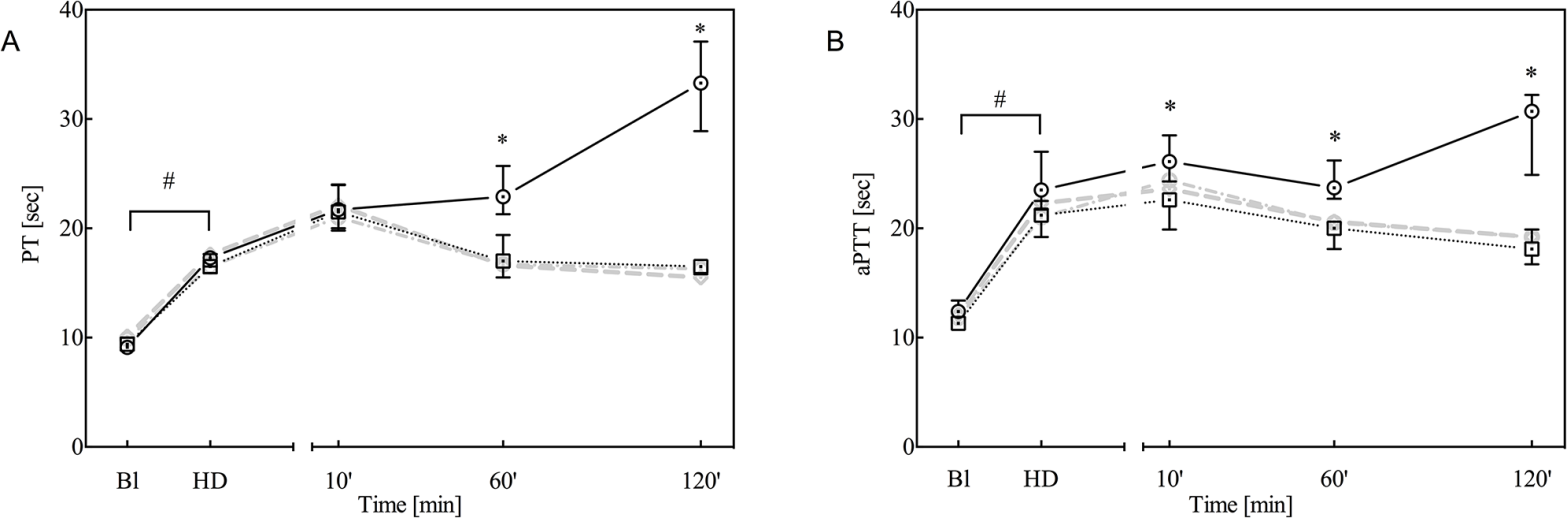
Prothrombin time (PT; A) and activated partial thromboplastin time (aPTT; B) at baseline (Bl), after haemodilution (HD) and at 10, 60 and 120 minutes after trauma. Data are presented as the median with inter-quartile ranges (IQR) for controls (solid black line, n = 7) and animals treated with 90 μg/kg rFVIIa (dotted black line, n = 7). For comparison, data from animals treated with either 180 or 360 μg/kg rFVIIa are presented as grey dotted lines. *P<0.05 control versus rFVIIa; #*P*<0.05 over time between baseline and haemodilution.

**Fig 3:**
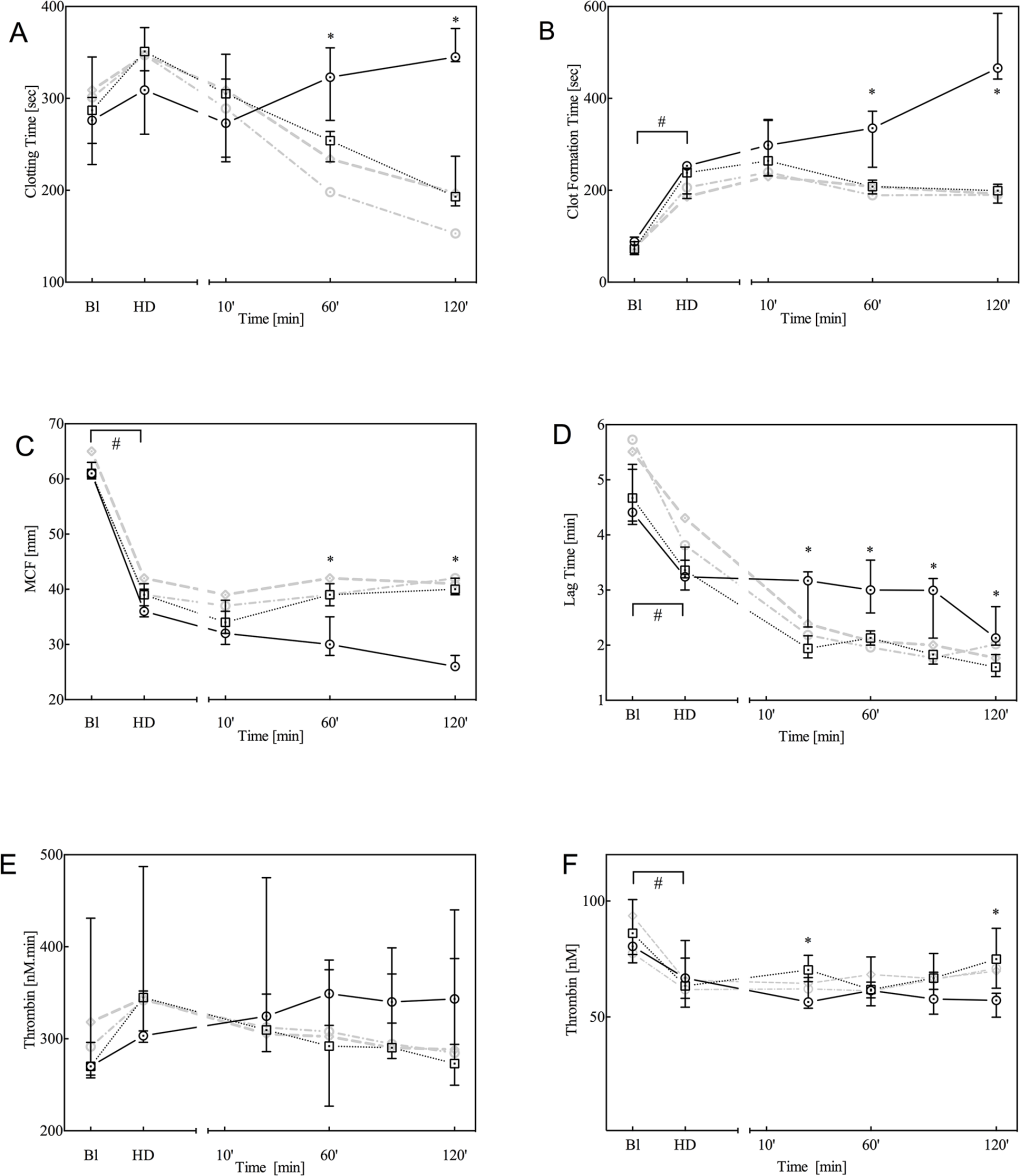
Thromboelastometry (TEM) analysis by means of ExTem (A, B and C) and plasma thrombin generation analysis (D, E, F; triggered by 0.25 pM TF) at baseline (Bl), after haemodilution (HD) and 10, 60 and 120 minutes after trauma. For TEM, the parameters clotting time (CT: A), clot formation time (CFT: B) and maximum clot firmness (MCF: C) are given, whereas for thrombin generation the lag time (D), endogenous thrombin potential (ETP; E) and peak height (F) are given. Data are presented as the median with inter-quartile ranges (IQR) for controls (solid black line, n = 7) and animals treated with 90 μg/kg rFVIIa (dotted black line, n = 7). For comparison, data from animals treated with either 180 or 360 μg/kg rFVIIa are presented as grey dotted lines. *P<0.05 control versus rFVIIa; #*P*<0.05 over time between baseline and haemodilution.

All baseline parameters were comparable among groups. As previously observed with this model [13,14], haemodilution produced a significant decrease in the concentration of erythrocytes, platelets, haemoglobin and fibrinogen (Fig 1A, 1B and 1C), whereas TAT levels remained unaltered (Fig 1D). Furthermore, haemodilution prolonged the PT and aPTT (Fig 2A and 2B). Whole-blood thromboelastometry parameters, determined by TEM analysis, indicated significantly decreased MCF and increased CFT (Fig 3C and 3B, respectively), whereas the dilution of coagulation factors resulted in a decrease in plasma thrombin generation (Fig 3E).

### Trauma

Following blunt liver trauma, the observed coagulopathy deteriorated, as indicated by further decreases in haemoglobin concentration, platelets and fibrinogen (Fig 1A, 1B and 1C: placebo), prolongation of the PT and aPTT (Fig 2A and 2B), and attenuation of thromboelastometry (Fig 3A–3C) at 120 minutes after trauma. Furthermore, TAT levels slightly increased over time, which indicates that trauma and rFVIIa induced activation of coagulation (Fig 1D).

### rFVIIa treatment following trauma

#### Blood loss

**Fig 4:**
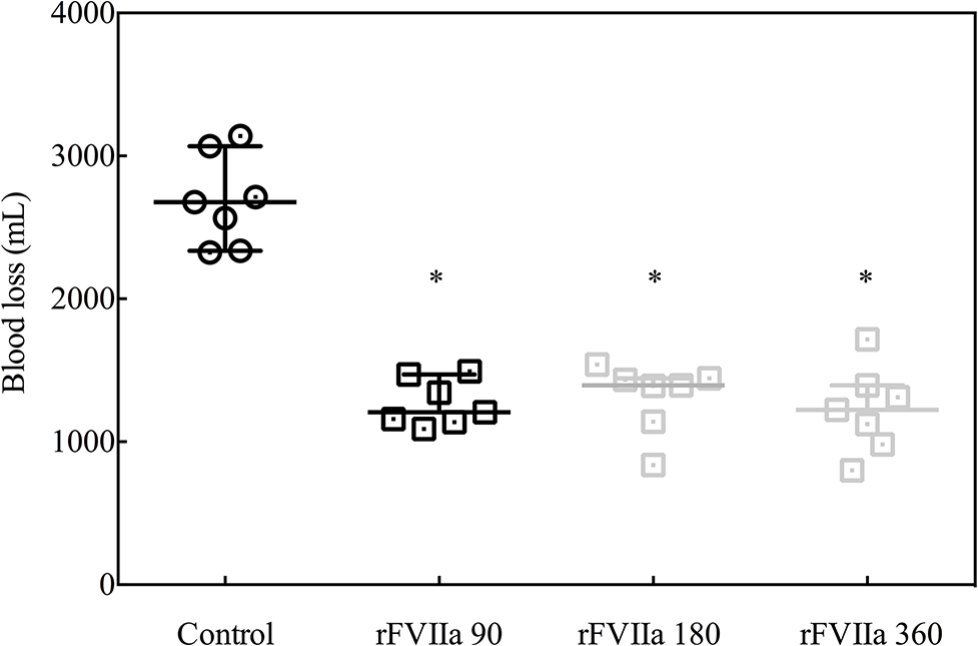
Total blood loss at the end of the observation period for animals not treated (control, black circles) or treated with 90 μg/kg (black squares), 180 μg/kg or 360 μg/kg rFVIIa. *P<0.05 rFVIIa versus control.

Blood loss was significantly lower in pigs treated with 90 μg/kg (1206 [1138–1470] mL), 180 μg/kg (1395 [1141–1444] mL) and 360 μg/kg rFVIIa (1223 [981–1394] mL) after liver trauma, relative to control animals (2677 [2337–3068)] mL, *P*<0.05 for all groups). The amount of blood loss was not influenced by the different concentrations of rFVIIa administered (Fig 4). Two of seven animals in the control group died before the end of the observation period, whereas all of the rFVIIa-treated animals survived (statistically not significant).

#### Coagulation parameters

To simplify data presentation and due to similarity among the three rFVIIa groups concerning all coagulation parameters, we focus only on data from the group treated with 90 μg/kg rFVIIa. Treatment with the two higher doses of rFVIIa had a similar impact on coagulation parameters and no differences were found among the three different doses of rFVIIa in the final data analyses.

Following administration of rFVIIa, haemoglobin and fibrinogen levels stabilised and remained elevated at 60 and 120 minutes after trauma, relative to the controls (Fig 1A and 1C). The number of platelets was significantly higher at 120 minutes after trauma relative to controls, whereas TAT levels were elevated in the rFVIIa treatment group relative to controls (Fig 1B and 1D). PT and aPTT were both reduced at 60 and 120 minutes after trauma and administration of rFVIIa.

#### Thromboelastometry analysis (TEM)

Thromboelastometry analysis (TEM) analysis revealed reduced clotting time and maximum CFT and increased MCF at 60 and 120 minutes after trauma in the rFVIIa treatment groups relative to placebo (Fig 3A–3C). On average (for the combined 60 and 120 minutes time points), clotting was accelerated by rFVIIa from 342 (323–361) to 234 seconds (192–258) for the clotting time and by almost 200 seconds from 403 (319–490) to 201 seconds (182–215) for the CFT.

#### Thrombin generation (TG)

**Fig 5:**
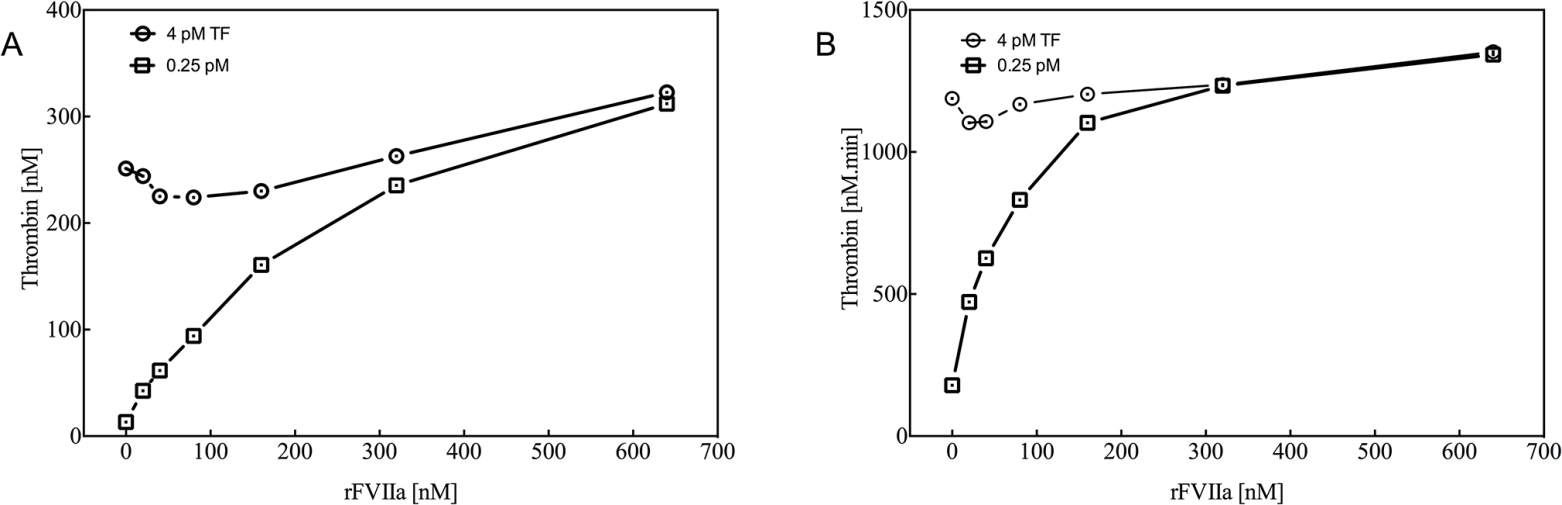
Contribution of rFVIIa to plasma thrombin generation assessed by the calibrated automated thrombogram and triggered with 0.25 (squares) or 4 (circles) pM tissue factor (TF) and 4 μM phospholipids. Left panel: Maximum thrombin generation presented as the peak height (A). Right panel: endogenous thrombin potential (ETP; B).

To assess whether the effect of rFVIIa treatment can be monitored by thrombin generation, the CAT assay was optimised with regard to phospholipid, TF and rFVIIa concentrations. rFVIIa was added to plasma obtained from healthy volunteers in increasing concentrations from 0 to 80 nM. Phospholipid concentrations varied between 0 and 6.4 μM, and the optimal concentration was 4 μM (data not shown). Varying the TF concentration between 0.25 and 4 pM revealed more pronounced thrombin generation by rFVIIa at 0.25 pM TF than at 4 pM TF, as indicated by peak height and ETP (Fig 5A and 5B). Based on these observations, thrombin generation in pig plasma samples was assessed using 0.25 pM TF and 4 μM phospholipids. After haemodilution, thrombin generation triggered by 0.25 pM TF was characterised by a shortening of the lag time (from 4.64 minutes [4.24–5.21] to 3.36 minutes [3.19–3.65], P<0.05) and a decrease in peak height from 81 nM (76–90) to 66 nM (58–75) (P<0.05), whereas the endogenous thrombin potential (ETP) was comparable between the two time points (270 nM [260–298] versus 318 nM [301–365], P = 0.06) (Fig 3D, 3E and 3F). Thrombin generation remained low after trauma and was comparable to that after haemodilution. The lag time decreased upon rFVIIa administration at 30, 60, 90 and 120 minutes after trauma. For the combined time points, the lag time shortened on average from 2.90 minutes (2.43–310) to 1.87 minutes (1.66–2.01) (all time points P<0.05). Despite a small significant difference in peak height between 30 and 120 minutes, the overall potential for thrombin generation (ETP) was not influenced by treatment with rFVIIa in trauma.

#### Macroscopic and microscopic tissue analysis

Post-mortem examination of the liver showed parenchymal injury with an average depth of 1.5–2 cm (70–90% of the total lobe depth). Immunostaining with vWF revealed laceration of venous vessels (up to 5 mm in diameter). There were no differences among the study groups. Histological slides from representative sections of the kidney, lung, liver and heart tissues showed no thromboembolic events in any of the animals. Immunostaining of lung tissue also indicated no evidence of thrombus formation (data not shown).

## Discussion

In this study, the effects of three dose levels of rFVIIa on coagulation parameters in a blunt traumatic liver injury model under severe hypothermia and haemodilution were assessed. rFVIIa treatment was effective in reducing trauma-induced blood loss even at a dose of 90 μg/kg, but with no further effect of increasing doses. These findings were supported by improved coagulation in TEM parameters and improved coagulation kinetics in thrombin generation, irrespective of rFVIIa dose.

Beneficial effects of rFVIIa treatment on coagulation parameters in a pig model of trauma were shown previously, but the effects of rFVIIa treatment on blood loss or survival were inconsistent [16–20]. The conflicting results in experimental studies of injury are likely the result of differences in study design and severity of injury. In this pig study, we demonstrated for the first time that 90 μg/kg rFVIIa is efficacious in combined blunt liver injury and coagulopathy in pigs, whereas previous studies used doses of 180–720 μg/kg to achieve beneficial effects [16–20]. We chose this minimum concentration due to current recommendations for clinically approved indications, such as haemophilia. In line with these recommendations, the present study showed that rFVIIa doses exceeding 90 μg/kg had no additional benefit on the primary endpoint of acute blood loss, or on most other analysed coagulation parameters in our experimental pig model. Because the efficacy of rFVIIa in major bleeding associated with complex coagulopathy also depends on other coagulation factors (e.g. fibrinogen), the combined effects of increased concentrations of rFVIIa and fibrinogen remain unclear [21].

Current plasma-based routine coagulation tests are not effective in determining or predicting the development of coagulopathy accurately. PT and aPTT show altered clotting times following trauma and have been shown to be independent risk factors for mortality; however, they are not closely associated with blood loss and are not considered suitable screening tests for coagulopathy [22–24]. Furthermore, both PT and aPTT only characterise the initiation of coagulation and do not reflect complete fibrin formation or shed light on anticoagulant mechanisms.

In this study, we compared for the first time PT, aPTT, TEM and thrombin generation in a trauma model following rFVIIa treatment. PT and aPTT were prolonged upon induction of trauma and shortened after rFVIIa treatment. Similarly, TEM analysis revealed prolonged CT and CFT, which was counteracted by rFVIIa treatment at 60 and 120 minutes after trauma, as shown previously [21]. Thrombin generation analysis indicated a shortened lag time and decreased peak height after induction of trauma, whereas the ETP was not altered after trauma. Furthermore, a strong effect on lag time was observed upon rFVIIa treatment, but ETP and peak height were hardly affected, which was also observed in previous studies in which FVIIa was shown to be a determinant of lag time but not of ETP or peak height [25]. Similarly, administration of rFVIIa was shown to not influence peak height or ETP in a computational model [26]. In contrast to these data, other studies demonstrated that rFVIIa had a marked impact on thrombin generation in haemophilic plasma [27]. This finding may be explained by an overall reduction of all coagulation factors caused by haemodilution and trauma. In the present study, neither TEM nor CAT was able to differentiate between different doses of rFVIIa, in accordance with the fact that no differences in haemostatic effect were observed between the three dose levels. However, to evaluate the usability of TEM or CAT in the present model, even lower doses of rFVIIa, with an intermediate effect on blood loss, should be included.

Because rFVIIa is a pro-coagulant protein, thromboembolic events are a major concern. However, in the present study, we did not observe an increased risk of adverse events, even with a high dose of 360 μg/kg. This is in contrast to a comparable study with PCC in the same porcine model: while 35 and 50 IU/kg PCC showed efficacy in reducing liver bleeding, 50 IU/kg PCC also increased the risk of thromboembolism and DIC. Thus, thromboembolism was found in all animals dosed with 50 IU/kg PCC, and 44% of these animals had signs of DIC [13]. PCCs contain either three or four coagulation factors (factors II, IX and X, with or without factor VII) and, depending on formulation, low doses of coagulation inhibitors such as protein C, protein S and heparin. For patients with life-threatening bleeding, rapid replacement of these coagulation factors is required, and PCCs serve as a concentrated source of the required coagulation factors. However, in these settings levels of coagulation inhibitors as well as procoagulants are usually decreased and the goal is to enhance thrombin generation and/or fibrin formation to promote clot formation. Depending on formulation, PCCs contain low doses of coagulation inhibitors such as protein C, protein S and heparin. Care must be taken to avoid the risk of thromboembolic complications because the levels of the of coagulation inhibitors care much lower than those of the coagulation factors. i.e. poorly balanced concentrations of pro- and anti-coagulant enzymes [28]. This hypothesis corresponds with in vitro and in vivo data that identified prothrombin overload as the most likely cause of thrombosis and disseminated intravascular coagulation [13,29,30]. Conclusively, the different mechanism on coagulation by rFVIIa (bypassing agent) and PCC (increase of several coagulation factors) might explain the lack of thromboembolic events following the application of rFVIIa [31,32].

This study has several limitations. First, to standardise the grade of coagulopathy, haemodilution had to be induced prior to infliction of injury. In clinical situations, however, haemodilution results from blood loss that is followed by large-volume infusion and administration of haemostatic agents upon coagulopathy. Second, as this study was performed in healthy, anaesthetised pigs, physiological responses such as pain and inflammation, which could affect haemostasis, were not evaluated. Several studies have shown that HES solutions induce coagulopathy caused by fibrinogen deficiency and dysfunction [33,34]. However, haemodynamic unstable patients with massive blood loss and severe haemorrhagic shock often require a combination of colloids and crystalloids for volume resuscitation. As all animals received comparable amounts of HES infusion during haemodilution, the impact on the coagulation system was comparable. Finally, the effect of human rFVIIa in pigs can be difficult to interpret, as human rFVIIa may have a reduced reactivity with pig coagulation factors and inhibitors, similar to what has been described in other species for human FVIIa and TF interaction [35].

In conclusion, rFVIIa treatment at a dose of 90 μg/kg reduced blood loss and improved various coagulation parameters in a pig model of liver trauma under haemodiluted and hypothermic conditions. No sign of adverse events was observed in the present study, and a dose-response was not observed (at the range of doses examined) even when increasing the dose four-fold compared with the efficacious concentration.
